# Increasing incidences and changes in treatment trends of clavicle fractures in adults during 2 decades in Denmark: a nationwide study on data from the Danish National Patient Registry

**DOI:** 10.2340/17453674.2025.43000

**Published:** 2025-01-28

**Authors:** Ida TRYGGEDSSON, Bjarke VIBERG, Per Hviid GUNDTOFT, Ilija BAN, Søren OVERGAARD, Tazio MALEITZKE, Arvind VON KEUDELL

**Affiliations:** 1 Department of Orthopaedic Surgery and Traumatology, Bispebjerg Hospital, Denmark; 2 Department of Orthopaedic Surgery and Traumatology, Odense University Hospital, Denmark; 3 Department of Orthopaedic Surgery and Traumatology, Hospital Lillebaelt Kolding, Denmark; 4 Department of Orthopaedic Surgery, Aarhus University Hospital, Denmark; 5 Department of Clinical Medicine, University of Copenhagen, Copenhagen, Denmark; 6 Trauma Orthopaedic Research Copenhagen Hvidovre (TORCH), Department of Orthopaedic Surgery, Copenhagen University Hospital – Amager and Hvidovre, Hvidovre, Denmark; 7 Charité – Universitätsmedizin Berlin, corporate member of Freie Universität Berlin and Humboldt-Universität Zu Berlin, Center for Musculoskeletal Surgery, Berlin, Germany; 8 Berlin Institute of Health at Charité – Universitätsmedizin Berlin, Julius Wolff Institute, Berlin, Germany; 9 Harvard Orthopaedic Trauma Initiative, Brigham and Women’s Hospital, Boston, USA

## Abstract

**Background and purpose:**

Clavicle fractures are common shoulder injuries, but treatment strategies are debated. While a non-surgical approach has been preferred historically, recent studies suggest surgical intervention may reduce non-union rates and improve outcomes for displaced fractures. Despite ongoing research there is still no consensus on the optimal treatment choice. We aimed to report national incidences and trends in treatment of clavicle fractures in Denmark across 2 decades.

**Methods:**

The Danish National Patient Register was used to extract data on clavicle fracture diagnosis and treatment in patients aged 18 and above from 1996 to 2018. Primary treatment was categorized as surgical if a relevant surgical procedure code was registered within 3 weeks of the fracture code, otherwise treatment was defined as non-surgical.

**Results:**

There were 81,597 clavicle fractures recorded; 67% were in men and the mean age was 50.3 years (standard deviation [SD] 19.5). The overall fracture incidence was 82/100,000/person-years, increasing by 11% from 76 in 1996 to 84 in 2018. The incidence was more than 50% higher in males (113) than females (53). 6,096 cases (7.5%) were treated surgically, mainly with plate osteosynthesis (94%). The surgical rate increased from 1% in 1996 to 14% in 2011, whereafter it decreased again. In 2018, 7% of clavicle fractures were treated surgically, with inter-hospital variations ranging from 0 to 15%.

**Conclusion:**

The incidence of clavicle fractures increased over the period. Non-surgical treatment remained prevalent, though surgical rates fluctuated with plate osteosynthesis being the preferred method.

Fracture of the clavicle is a common injury, often resulting from sports, high-energy trauma, or same-height falls, thus affecting both younger and older patients [1]. Optimal treatment of clavicle fractures has been and still is the subject of debate among clinicians. Historically, these fractures have been treated non-surgically, but at the turn of the millennium several studies opposed this approach, and recommended surgical treatment of displaced fractures [2,3]. In the past 2 decades, several randomized studies have been reporting divisive results, some advocating for surgical treatment and some for non-surgical treatment [4,5]. While there is still no consensus on optimal management, most clavicle fractures are treated non-surgically, but studies show an increase in surgically treated clavicle fractures over time [6,7]. Previous studies on clavicle fracture incidence have been based on smaller cohorts, limited geographically or by selected patient groups, or covering only a few years [8-10]. The Danish National Patient Registry (DNPR) provides longitudinal registration of diagnoses and treatments with complete nationwide coverage, making Denmark an ideal place for epidemiological studies [11]. By utilizing the strengths of the DNPR, this research seeks to fill the gap in knowledge regarding incidences on a national level along with long-term trends in treatment practices for clavicle fractures. We aimed to report the national incidences of clavicle fractures and trends in treatment in Denmark from 1996 to 2018.

## Methods

### Study design

This is a nationwide register study on clavicle fracture incidences and treatments in adult patients from 1996 to 2018 with data from the Danish National Patient Register (DNPR). This study adheres to “The REporting of studies Conducted using Observational Routinely-collected health Data” (RECORD) guideline [12].

### Setting

In Denmark, all permanent residents are registered in the Civil Registration System with a unique and unchangeable personal identification number [13]. This number allows linkage on an individual level across all national registers until a resident dies or emigrates.

The Danish healthcare system is tax-funded, ensuring free public healthcare for all citizens, including emergency treatment, hospital care, and outpatient visits. Acute fractures are exclusively managed in the public emergency rooms or outpatient clinics. When a patient has an encounter with the healthcare system, the involved department is required to record the injury diagnosis in the electronic patient file [11]. If the patient receives surgical treatment, it will be registered with a procedure code. Diagnosis and procedure codes are automatically transferred to the DNPR.

### Data source

The DNPR is a comprehensive database that contains detailed records of all hospitalizations, outpatient visits, and emergency room visits in Denmark. The register has had national coverage on hospital admissions since 1978 and on emergency visits since 1995. The DNPR holds data from public hospitals on diagnoses, treatments, and patient demographics for all patient contacts that received tax-supported treatment. The register is used for research, healthcare planning, and monitoring public health trends [11]. Since 1994, diagnoses have been registered according to the International Statistical Classification of Diseases version 10 (ICD-10) [14], while procedures have been coded according to the Danish version of the Nordic Medico-Statistical Committee Classification of Surgical Procedures (NOMESCO) since 1996 [15].

Data on the total population and subgroups (sex and age) were obtained from Statistics Denmark, which maintains data on the number of citizens in Denmark [16].

### Data quality and bias

The coding accuracy and data validity within the DNPR, measured by the positive predictive value (PPV), has been assessed to a PPV of 83% for correct primary diagnosis overall [11]. Given the precision of radiographic diagnoses, fracture coding in the DNPR generally has a high PPV, as seen in validation studies for humeral and ankle fractures with PPVs of 89% [17,18]. We expect a similar PPV for clavicle fractures and minimal information bias due to coding inaccuracy. Potential miscoding is assumed to be consistent over the study period, not affecting variations over time.

The universal healthcare system in Denmark, along with nationwide coverage of both hospital admissions and emergency visits, ensures high completeness and reduces the impact of selection bias related to income, health insurance, or hospital location, making Denmark an ideal setting for register studies.

### Participants

We included patients aged 18 years and older diagnosed with a clavicle fracture (ICD-10: S42.0) during the study period. To ensure accuracy and prevent multiple registrations of the same injury, a 90-day diagnosis quarantine period for the diagnosis code S42.0 was implemented. Clavicle fractures coded within 90 days of the primary clavicle fracture were excluded from the dataset. After 90 days, the same patient could be included with a new clavicle fracture.

Each patient was categorized as receiving surgical or non-surgical treatment based on procedure codes according to NOMESCO. Surgical treatment was defined as the registration of a relevant procedure code within 3 weeks of the injury. Patients without a relevant procedure code within this timeframe or coded with a procedure later than 3 weeks after the injury were classified as receiving primary non-surgical treatment.

### Variables

The study spans from 1996 to 2018. The application for use of data from the DNPR was made in 2019, explaining the end of the study period, while 1996 marks the first full year of nationwide coverage of emergency visit in the DNPR. Sex and age of each patient were determined by their social security number and date of diagnosis. Sex was categorized as male or female. Age groups were divided into 20-year intervals (18–39, 40–59, 60–79, and ≥ 80) to reflect different lifestyles and physiological patterns. The relevant procedure codes that defined surgical treatment included plate osteosynthesis (KNBJ62), intramedullary nail osteosynthesis (KNCJ52), K-wire osteosynthesis (KNBJ42), screw osteosynthesis (KNBJ82), combined methods (KNBJ82), or unspecified method (KNBJ92).

### Statistics

We analyzed the data using STATA version 17 (StataCorp LLC, College Station, TX, USA). To report on age, sex, number of clavicle fractures, and treatment, we used descriptive statistics presented in numbers and percentages. The average age is calculated as a weighted average based on the midpoint of age groups with 5-year intervals. The incidences were calculated as the number of clavicle fractures per 100,000 persons per year (person-years). We based our calculations on the entire adult population in Denmark; hence no sample estimates and no calculations of confidence intervals are provided.

### Data access, ethics, data sharing, use of AI, funding, and disclosures

Data approval was obtained by the Region of Southern Denmark (jr.nr 20/187). Data was stored on “Forskermaskinen,” a research database at the Danish Health Data Authority, with authors PHG and BV having full access to available data and providing the dataset to the rest of the authors. Ethical approval was not needed for this study according to Danish legislation. No funding was obtained for this study. We acknowledge using ChatGPT (OpenAI) and Grammarly to prepare this manuscript. ChatGPT provided inspiration for the content and structure of the discussion section. Grammarly was utilized to enhance sentence structure, and correct grammatical errors. The authors thoroughly reviewed and edited all content and suggestions provided by these tools to ensure accuracy, coherence, and alignment with the research objectives.

None of the authors have conflicts of interest to declare related to this article. Complete disclosure of interest forms according to ICMJE are available on the article page, doi: 10.2340/17453674.2025.43000

## Results

### Epidemiology

**Figure 1:**
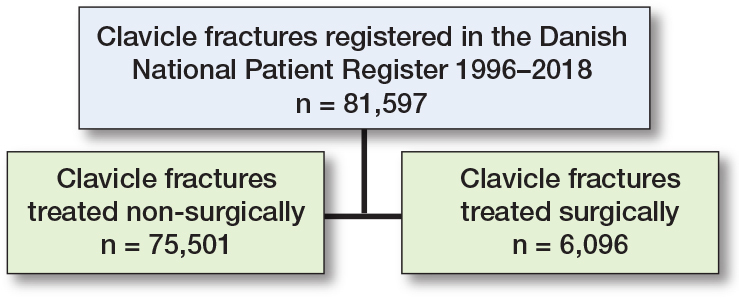
Flowchart of patient distribution.

**Figure 2:**
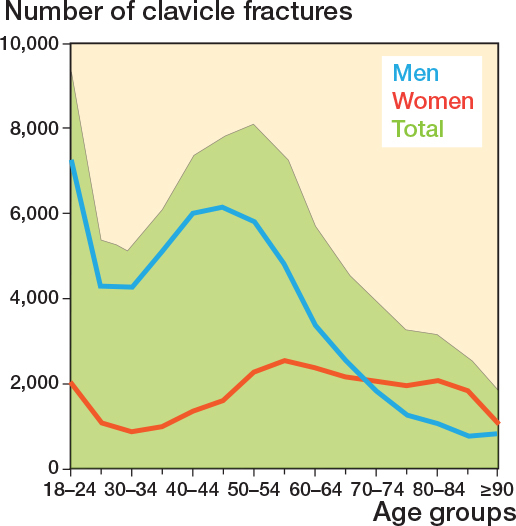
Number of clavicle fractures distributed by sex and age groups.

Between 1996 and 2018, 81,597 clavicle fractures were registered in the DNPR (Table 1 and Figure 1). Most clavicle fractures occurred in men (54,839; 67%) compared with women (26,758; 33%) (Figure 2). The mean age for patients with a clavicle fracture was 50.3 years (standard deviation [SD] 19.5), and the average age was 45.7 years for men (SD 17.2) and 59.6 years (SD 20.6) for women. Over the study period, there was a 23% increase in the total number of clavicle fractures, rising from 3,156 in 1996 to 3,885 in 2018. During the same period, the adult population in Denmark increased by 11%, from 4.1 million to 4.6 million people. Most fractures were sustained by males in age groups below 60 years (Figure 2). Additional information on the number of fractures and incidences by year is provided in Supplementary Tables S1 and S2.

**Table 1:** Demographics and treatment of the cohort (n = 81,597) reported as number of fractures and percentage (%) of fractures in total and each age group

Factor	Total n = 81,597	Age 18–39 n = 25,887	Age 40–59 n = 30,499	Age 60–79 n = 17,584	Age ≥ 80 n = 7,627
Sex					
Female	26,758 (33)	5,008 (19)	7,790 (26)	8,572 (49)	5,388 (71)
Male	54,839 (67)	20,879 (81)	22,709 (74)	9,012 (51)	2,239 (29)
Treatment					
Surgical	6,096 (7.5)	2,536 (9.8)	2,865 (9.4)	675 (3.8)	20 (0.3)
Non-surgical	75,501 (92)	23,351 (90)	27,634 (91)	16,909 (96)	7,607 (100)

**Figure 3:**
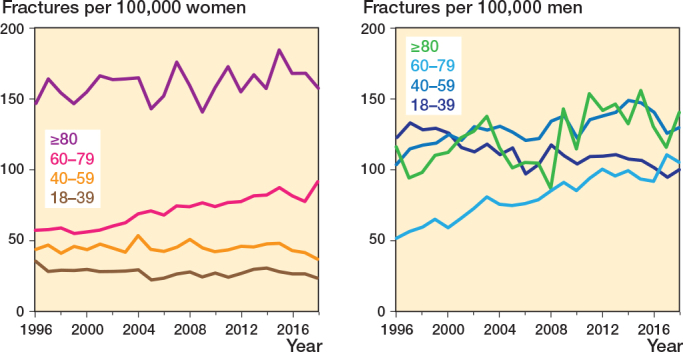
Trends in incidence of clavicle fractures over time stratified by sex and age groups. Incidence is calculated as number of fractures per 100,000 persons in the age group.

The overall incidence for the study period was 82 clavicle fractures per 100,000 person-years. There was an 11% increase from 76 clavicle fractures per 100,000 persons in 1996 to 84 clavicle fractures per 100,000 persons in 2018. The incidence per year for males (113 fractures per 100,000 person-years) was higher than for females (53 fractures per 100,000 person-years). Among women, the highest incidence occurred in the age group ≥ 80 years (161 per 100,000 person-years) (Figure 3). The highest incidence for males was found in the age group 40–59 years (129 per 100,000 person-years), and the incidence increased by 25% for this group during the study period. However, the most notable increase in incidence was observed in males 60–79, with a 103% increase from 1996 to 2018 (Figure 3).

### Treatment

**Figure 4:**
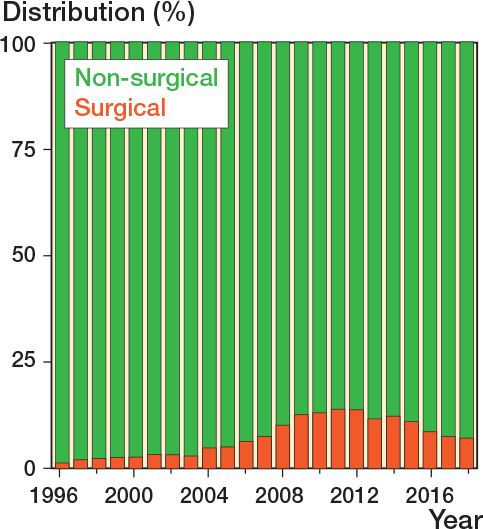
Distribution (percentage) of surgical and non-surgical treatment over time.

**Figure 5:**
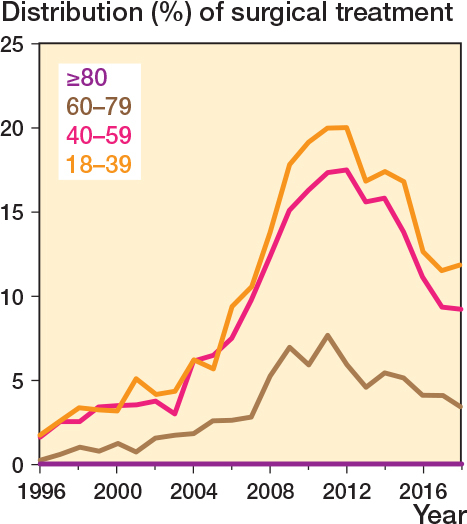
Distribution (percentage) of surgical treatment over time stratified by age groups.

**Figure 6:**
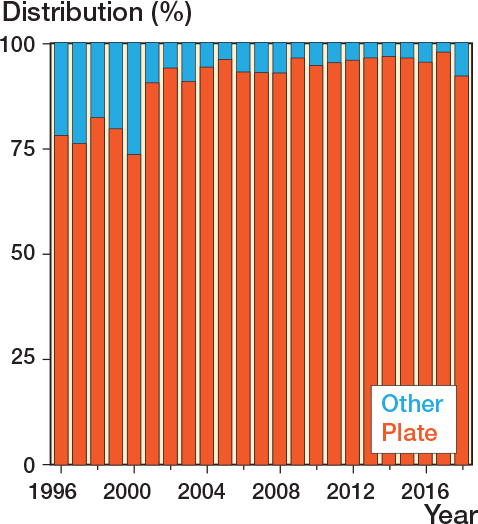
Percentage of type of surgical treatment over time stratified by plate and other (including intermedullary nail, screws alone, combined method, or unspecified method).

Most fractures were treated non-surgically (75,501; 93%) compared with surgically (6,096; 7%). The percentage surgically treated increased from 1% in 1996, peaked at 14% in 2011, and gradually declined to 7% in 2018 (Figure 4). Surgical treatment was predominantly performed in younger patient groups (18–59) (Figure 5). Plate osteosynthesis was the most commonly used surgical method (5,761; 94%) (Figure 6). In 6% of cases, intramedullary nail osteosynthesis, screw osteosynthesis, combined methods, or an unspecified method was applied. Only for the year 2018 were we able to report on surgical rates for orthopedic departments across Denmark (Table 2). The lowest surgical rate reported was 0% (0 surgical cases out of 89 clavicle fractures), while the highest was 15% (29 out of 168).

**Table 2:** Numbers and percentages of surgically and non-surgically treated clavicle fractures in Denmark in 2018: stratification by regions and hospitals as they were named in 2018. Values are count (%)

Region and hospital	Surgery	Non-surgery
North Denmark Region	32 (9.9)	323 (91)
Nordjylland	0 (0)	89 (100)
Aalborg	32 (12)	234 (88)
Central Denmark Region	101 (10)	984 (90)
Randers	11 (7.3)	139 (93)
Holstebro	18 (8.5)	194 (92)
Viborg	29 (15)	168 (85)
Aarhus	10 (4.1)	235 (96)
Kolding	21 (12)	124 (88)
Horsens	12 (8.8)	124 (91)
Region of Southern Denmark	48 (8.7)	549 (91)
Sydvestjysk	17 (13)	114 (87)
Soenderjylland	12 (7.4)	150 (93)
Odense	19 (6.3)	285 (94)
Region Zealand	26 (5.3)	469 (95)
Zealand University Hospital	26 (5.3)	469 (95)
Capital Region of Denmark	62 (5.4)	1,143 (95)
Nordsjaelland	20 (8.2)	225 (92)
Herlev and Gentofte	10 (3.6)	269 (94)
Hvidovre and Amager	16 (5.3)	284 (95)
Bispebjerg and Frederiksberg	3 (1.2)	247 (99)
Rigshospitalet and Glostrup	9 (10)	81 (90)
Bornholm	4 (9.8)	37 (90)
Unspecified hospitals	12 (6.5)	172 (93)
Total	281 (7.2)	3,604 (93)

## Discussion

We aimed to provide an overview on variations of incidences and management of clavicle fractures over 2 decades. We observed an increase in incidences over time, changes in age distribution, and fluctuations in surgical versus non-surgical treatment. The data reveal that although non-surgical treatment remained the most frequently used approach, surgery rates increased over the period, with a notable peak in 2011.

We observed an incidence rate of 82 clavicle fractures per 100,000 person-years. Previous studies from 1988 to 2012 reported slightly lower incidence rates, ranging from 29 fractures per 100,000 person-years in Scotland to approximately 60 in the United States and Sweden [7-9].

Several studies have reported on the incidence of clavicle fractures in single cities or limited areas but, to our knowledge, only 2 other studies have reported on national incidences of clavicle fractures [6,7]. A study from Sweden assessed data from the national Swedish Hospital Discharge Register through 2001–2012 and found an increase in the incidence of clavicle fractures from 36/100,00/year in 2001 to 59/100,000/year in 2012, a 64% increase in incidence. For the same years, we report a 11% increase in incidence from 76 to 84/100,00/year. While the Swedish register has an almost 100% coverage of inpatient visits, the coverage of hospital-based outpatient care is reported to be lower, which may cause a missed number of non-surgically treated clavicle fractures [19]. This may explain part of the lower incidence in Sweden over the period. As clavicle fractures are often related to sports or traffic injuries, especially bicycle accidents [1], a well-developed biking infrastructure in Denmark may possibly cause more bicycle-related injuries, hence more clavicle fractures, compared with neighboring countries. However, this is a hypothesis, since we were not able to report on injury mechanism.

Another nationwide register-based study was conducted in Finland covering the years from 1987 to 2010 [6]. Huttunen et al. analyzed data from the Finnish Discharge Register and reported a ninefold increase in the number of surgically treated clavicle fractures over the study period. The Finnish register contains data only on inpatient care, but no outpatient visits, therefore the actual incidence of clavicle fractures was not known, but they still assumed that the proportion of surgically treated patients had increased. Including both inpatient and outpatient visits, we found the proportion of surgically treated clavicle fractures increasing from 1% in 1996 to 7% in 2018.

Our findings align with results from the Swedish study reporting 10% of patients being treated surgically in 2001–2012 [7].

We found the frequency of surgical intervention peaked at 14% in 2012 then declined to 7%, which is similar to trends reported in the Swedish and Finnish studies during the same years.

Changes may be explained by evolving evidence at the time. In the early 1990s, the traditional non-surgical management was questioned due to reports of high non-union rates and decreased functionality [2]. In 2007, a multicenter randomized controlled trial (RCT) conducted by the Canadian Orthopaedic Trauma Society found that plate fixation led to faster healing, lower non-union rates, and better functional outcomes, hence supporting surgery for displaced fractures in active adults [4]. However, later trials found no long-term differences between surgical and non-surgical treatment. Looking at midshaft fractures, Robinson et al. (2012), Virtanen et al. (2012), and Qvist et al. (2018) all reported that while surgery reduced non-union rates, long-term outcomes were equally good for both treatments [5,20,21].

Axelrod et al. (2020), echoed these findings in a systematic review, concluding that surgical treatment did not improve long-term functional scores to a degree that patients would consider clinically important [22].

The optimal treatment for clavicle fractures remains a subject of ongoing debate, and no definitive conclusions have yet been established. However, more studies have shown no difference in long-term functional outcomes, which likely contributed to a decline in primary surgical rates internationally. In Denmark, rates may have also been affected by an official clinical practice recommendation by the Danish Orthopaedic Society in 2012 that favored non-surgical treatment for displaced midshaft clavicle fracture based on a comprehensive review of available evidence [23].

Throughout the study, we found that more than 75% of surgical cases were managed with plate fixation, increasing to 98% in recent years, as shown in Figure 5. This trend is in line with favorable results for the use of locking plates. For instance, a biomechanical study from 2008 found clavicle locking plates superior to non-locking plates in terms of load and bending resistance [24]. Additionally, Fridberg et al. (2013) retrospectively assessed more than 100 cases of clavicle fractures and reported advantages of locking plates despite a 5% failure rate and one-third needing implant removal [25]. The availability of better implants and supportive literature possibly influenced the preference for plate fixation and increased surgical rates.

In addition to overall data, we were able to retrieve information on inter-hospital differences in surgical rates for the single year of 2018. On average, 7% of clavicle fractures were treated with surgery in 2018, though this varied between hospitals, from 1 hospital reporting zero surgical cases out of 89 clavicle fractures, and another hospital treating 29 out of 168 fractures (15%) surgically.

To our knowledge, previous register-based studies have not included this kind of information. Data may indicate varying treatment criteria between hospitals, which was also mentioned by Ban et al. in a questionnaire-based study performed in Denmark, Sweden, and Finland in 2012–2014 [26]. Assessing differences in treatment preferences among surgeons in public hospitals, they found 80% of participating hospitals preferred surgical treatment for displaced clavicle fractures despite evidence at the time not supporting routine surgical treatment. The study highlighted a discrepancy between clinical practice and evidence-based recommendations, as well as different criteria for allocation to surgical treatment among hospitals.

However, our results of varying surgical rates could also be explained by smaller hospitals referring clavicle patients to better equipped orthopedic or shoulder-specific departments within their region. The proximity of a hospital to a big city may also impact the number of surgical cases, as urban hospitals often encounter a higher volume of high-energy injuries related to busy traffic, city nightlife, sports, and recreational events, and generally serve a younger population with high levels of physical activities and risk of injuries. However, we cannot reject the possible element of discrepancies in surgeon or hospital preferences, which underlines the lack of an evidence-based guideline for the optimal treatment of these fractures that still does not exist.

While the typical clavicle fracture patient is often a young, active male, our study showed a higher incidence of fractures present in males aged 40–59. While age-related frailty and decreased bone density may explain the high incidence reported for the oldest age groups, more fractures among the middle-aged may be due to the higher engagement in physical activities observed in recent years [27]. Unfortunately, we could not report on injury mechanisms due to the limitations of the register data. However, most clavicle fractures in younger patients occur in high-energy trauma, often caused by traffic accidents or sports. Active patients in the age group 40–60 are likely young and healthy and may prefer surgery to achieve a quicker return to a pre-injury activity level. Hence, patient demands may have also affected increasing surgical rates, as suggested in the register-based study from Finland, which also reported an increase in surgically treated clavicle fractures among younger and middle-aged patients [6].

### Limitations

First, the DNPR does not provide details on specific clavicle fracture types, such as medial, midshaft, or lateral fractures, nor does it distinguish between displaced and undisplaced fractures. This lack of detail limits our ability to analyze treatment trends for individual subcategories, particularly displaced fractures, where the debate on surgical versus non-surgical treatment is most prominent. As a result, we are unable to provide the clinically relevant insights that such differentiation would offer. In addition, the DNPR also lacks information on functional outcomes, quality of life, injury mechanism, and patient comorbidities, hence limiting our assessment of how these factors may influenced the incidences and treatment approaches. Second, the lack of validation of the clavicle diagnosis in the DNPR is also a limitation of this study, but the coding of fractures in the DNPR has a generally high accuracy and likely remained the same over the study period. Third, we chose 3 weeks as a cut-off between primary and secondary treatment, knowing some may argue this timeframe should have been shorter or longer, which would have affected the number of surgical cases. We argue that if surgery was the primary choice of treatment, it should be performed “acutely” within 3 weeks from injury, while if surgery was performed later, the fracture was initially planned to be treated non-surgically.

### Strengths

First, we included a large and comprehensive sample size, which enhances reliability of the findings. Additionally, the universal healthcare system in Denmark reduces the impact of selection bias related to income, health insurance, or hospital location, making it an ideal setting for register studies. Second, the use of longitudinal data collected over many years allows for the analysis of trends and variations over time, making the findings highly relevant to actual clinical practices. Third, the DNPR has high completeness, as data is included from all hospitals and clinics across the nation, ensuring nationwide coverage. This provides a complete picture of the incidence and treatment patterns that are representative of an entire population.

### Conclusion

We found an overall incidence of 82 clavicle fractures per 100,000 person-years with 2 out of 3 fractures happening in males. The incidence increased 10.5% over the period, particularly among men aged above 40 and women above 60. Non-surgical treatment remained the preferred treatment across all age groups, though the surgical rate increased from 1% to 7%, including a temporary peak at 14% in 2011 followed by a decline in subsequent years. The fluctuations suggest a dynamic interplay between clinical practice, available evidence, guidelines, and demographics.  

## Supplementary Material



## Supplementary data

Tables S1 and S2 are available as supplementary data on the article page, doi: 10.2340/17453674.2025.43000

## Notes

IT, IB, PG, BV came up with the idea for the study. The dataset was provided by PG and BV. IT conducted data analyses and wrote the draft of the manuscript. All authors contributed to developing and refining the manuscript.

Handling co-editor: Taco Gosens

*Acta* thanks Jan Erik Madsen and Michael Schneider for help with peer review of this manuscript.
