# Supplementary material for: Increasing incidences and changes in treatment trends of clavicle fractures in adults during 2 decades in Denmark: a nationwide study on data from the Danish National Patient Registry
Source: Acta Orthop. 2025 Jan 28;96:135–41. doi: 10.2340/17453674.2025.43000 (PMC11783182; doi:10.2340/17453674.2025.43000)
Supplement: Supplementary file 1 [file ActaO-96-43000-s1.pdf]

# Supplementary data

## Table S1. Number of clavicle fractures from 1996 to 2018

| | Total | 1996 | 1997 | 1998 | 1999 | 2000 | 2001 | 2002 | 2003 | 2004 | 2005 | 2006 | 2007 | 2008 | 2009 | 2010 | 2011 | 2012 | 2013 | 2014 | 2015 | 2016 | 2017 | 2018 |
|---|---|---|---|---|---|---|---|---|---|---|---|---|---|---|---|---|---|---|---|---|---|---|---|---|
| **Total** | 81,597 | 3,156 | 3,325 | 3,287 | 3,353 | 3,352 | 3,309 | 3,380 | 3,441 | 3,494 | 3,343 | 3,175 | 3,370 | 3,631 | 3,624 | 3,436 | 3,700 | 3,792 | 3,888 | 3,982 | 4,043 | 3,853 | 3,778 | 3,885 |
| **Sex** | | | | | | | | | | | | | | | | | | | | | | | | |
| Female | 26,758 | 1,066 | 1,061 | 1,022 | 1,033 | 1,040 | 1,081 | 1,078 | 1,071 | 1,198 | 1,057 | 1,065 | 1,184 | 1,218 | 1,148 | 1,163 | 1,199 | 1,223 | 1,294 | 1,318 | 1,386 | 1,292 | 1,271 | 1,290 |
| Male | 54,839 | 2,090 | 2,264 | 2,265 | 2,320 | 2,312 | 2,228 | 2,302 | 2,370 | 2,296 | 2,286 | 2,110 | 2,186 | 2,413 | 2,476 | 2,273 | 2,501 | 2,569 | 2,594 | 2,664 | 2,657 | 2,561 | 2,507 | 2,595 |
| **Age** | | | | | | | | | | | | | | | | | | | | | | | | |
| 18–39 | 26,270 | 1,373 | 1,407 | 1,364 | 1,371 | 1,340 | 1,219 | 1,177 | 1,213 | 1,139 | 1,097 | 953 | 1,017 | 1,125 | 1,061 | 1,020 | 1,048 | 1,068 | 1,095 | 1,087 | 1,071 | 1,026 | 989 | 1,010 |
| 40–59 | 30,933 | 1,066 | 1,177 | 1,175 | 1,229 | 1,277 | 1,285 | 1,343 | 1,311 | 1,428 | 1,329 | 1,277 | 1,318 | 1,445 | 1,446 | 1,296 | 1,398 | 1,447 | 1,468 | 1,548 | 1,551 | 1,458 | 1,341 | 1,320 |
| ≥60 | 25,331 | 740 | 768 | 779 | 788 | 787 | 844 | 895 | 962 | 971 | 960 | 991 | 1,094 | 1,109 | 1,190 | 1,171 | 1,296 | 1,319 | 1,361 | 1,378 | 1,463 | 1,395 | 1,490 | 1,580 |
| 18–24 | 9,302 | 457 | 474 | 437 | 426 | 407 | 374 | 386 | 379 | 329 | 343 | 324 | 347 | 409 | 380 | 392 | 416 | 425 | 460 | 451 | 436 | 426 | 395 | 429 |
| 25–29 | 5,385 | 324 | 312 | 293 | 298 | 289 | 259 | 231 | 256 | 259 | 248 | 187 | 210 | 193 | 184 | 160 | 195 | 209 | 186 | 202 | 248 | 187 | 223 | 232 |
| 30–34 | 5,139 | 270 | 293 | 324 | 293 | 284 | 269 | 241 | 238 | 236 | 238 | 195 | 198 | 226 | 215 | 200 | 178 | 191 | 197 | 194 | 156 | 187 | 165 | 151 |
| 35–39 | 6,061 | 313 | 310 | 295 | 334 | 333 | 297 | 302 | 318 | 298 | 255 | 231 | 238 | 279 | 251 | 253 | 239 | 227 | 242 | 229 | 217 | 217 | 194 | 189 |
| 40–44 | 7,334 | 291 | 361 | 341 | 323 | 338 | 341 | 343 | 335 | 375 | 329 | 322 | 352 | 364 | 347 | 270 | 287 | 312 | 310 | 315 | 309 | 294 | 252 | 223 |
| 45–49 | 7,747 | 315 | 328 | 301 | 289 | 354 | 318 | 354 | 309 | 345 | 317 | 324 | 320 | 349 | 377 | 339 | 417 | 406 | 354 | 399 | 350 | 337 | 265 | 280 |
| 50–54 | 8,079 | 284 | 297 | 316 | 352 | 300 | 325 | 318 | 311 | 331 | 327 | 329 | 313 | 380 | 369 | 333 | 363 | 345 | 416 | 426 | 432 | 376 | 410 | 426 |
| 55–59 | 7,339 | 165 | 183 | 203 | 253 | 265 | 282 | 312 | 339 | 356 | 335 | 280 | 305 | 331 | 319 | 327 | 313 | 363 | 367 | 392 | 438 | 439 | 391 | 381 |
| 60–64 | 5,757 | 124 | 128 | 135 | 137 | 154 | 158 | 181 | 198 | 240 | 261 | 259 | 298 | 311 | 318 | 294 | 288 | 310 | 319 | 321 | 298 | 319 | 357 | 349 |
| 65–69 | 4,704 | 88 | 111 | 125 | 131 | 108 | 115 | 132 | 140 | 137 | 162 | 150 | 185 | 221 | 236 | 244 | 280 | 305 | 309 | 313 | 309 | 284 | 296 | 323 |
| 70–74 | 3,908 | 129 | 114 | 128 | 125 | 101 | 131 | 120 | 150 | 140 | 128 | 138 | 138 | 140 | 152 | 167 | 179 | 205 | 213 | 218 | 264 | 244 | 279 | 305 |
| 75–79 | 3,226 | 116 | 123 | 108 | 110 | 124 | 115 | 131 | 141 | 123 | 114 | 132 | 125 | 125 | 154 | 132 | 165 | 147 | 143 | 173 | 170 | 167 | 180 | 208 |
| 80–84 | 3,142 | 127 | 150 | 132 | 129 | 124 | 155 | 145 | 133 | 118 | 118 | 119 | 137 | 107 | 132 | 129 | 148 | 130 | 151 | 142 | 170 | 141 | 145 | 160 |
| 85–89 | 2,603 | 104 | 92 | 91 | 90 | 107 | 101 | 105 | 122 | 117 | 89 | 109 | 125 | 113 | 109 | 97 | 133 | 124 | 127 | 121 | 131 | 138 | 130 | 128 |
| ≥90 | 1,871 | 49 | 49 | 58 | 63 | 64 | 69 | 79 | 72 | 90 | 79 | 76 | 79 | 83 | 81 | 99 | 99 | 93 | 94 | 86 | 115 | 97 | 96 | 101 |

## Table S2. Fracture incidence per 100,000 persons per year in Denmark from 1996 to 2018

| | 1996 | 1997 | 1998 | 1999 | 2000 | 2001 | 2002 | 2003 | 2004 | 2005 | 2006 | 2007 | 2008 | 2009 | 2010 | 2011 | 2012 | 2013 | 2014 | 2015 | 2016 | 2017 | 2018 |
|---|---|---|---|---|---|---|---|---|---|---|---|---|---|---|---|---|---|---|---|---|---|---|---|
| **Total (female + male)** | 76 | 80 | 79 | 80 | 80 | 79 | 81 | 82 | 83 | 79 | 75 | 80 | 85 | 84 | 80 | 85 | 87 | 88 | 89 | 90 | 85 | 82 | 84 |
| **Female** | | | | | | | | | | | | | | | | | | | | | | | |
| Total (age ≥18) | 50 | 50 | 48 | 48 | 49 | 51 | 50 | 50 | 56 | 49 | 50 | 55 | 56 | 53 | 53 | 54 | 55 | 58 | 58 | 61 | 56 | 55 | 55 |
| Age 18–39 | 36 | 29 | 30 | 30 | 30 | 29 | 29 | 29 | 30 | 23 | 24 | 27 | 28 | 25 | 27 | 25 | 27 | 30 | 31 | 28 | 27 | 27 | 24 |
| Age 40–59 | 44 | 47 | 42 | 46 | 44 | 48 | 45 | 42 | 54 | 44 | 43 | 46 | 51 | 46 | 42 | 44 | 46 | 46 | 48 | 49 | 43 | 42 | 37 |
| Age 60–79 | 57 | 58 | 59 | 56 | 56 | 58 | 60 | 63 | 69 | 71 | 68 | 74 | 74 | 77 | 74 | 77 | 78 | 82 | 82 | 88 | 82 | 78 | 92 |
| Age ≥80 | 147 | 164 | 154 | 147 | 155 | 167 | 164 | 165 | 165 | 144 | 152 | 176 | 160 | 141 | 158 | 173 | 155 | 168 | 158 | 184 | 169 | 169 | 158 |
| **Male** | | | | | | | | | | | | | | | | | | | | | | | |
| Total (age ≥18) | 103 | 111 | 111 | 113 | 113 | 109 | 112 | 115 | 112 | 111 | 102 | 105 | 115 | 117 | 107 | 117 | 119 | 120 | 122 | 120 | 114 | 111 | 114 |
| Age 18–39 | 123 | 133 | 129 | 130 | 127 | 116 | 113 | 119 | 111 | 116 | 98 | 104 | 118 | 110 | 105 | 110 | 110 | 111 | 108 | 107 | 102 | 95 | 100 |
| Age 40–59 | 104 | 115 | 118 | 119 | 125 | 121 | 131 | 128 | 131 | 127 | 121 | 123 | 135 | 138 | 123 | 136 | 139 | 141 | 149 | 148 | 141 | 126 | 130 |
| Age 60–79 | 52 | 57 | 60 | 65 | 60 | 66 | 73 | 81 | 76 | 75 | 76 | 79 | 85 | 91 | 86 | 94 | 101 | 96 | 100 | 94 | 92 | 111 | 106 |
| Age ≥80 | 116 | 95 | 99 | 111 | 113 | 123 | 128 | 138 | 116 | 102 | 106 | 105 | 87 | 143 | 115 | 154 | 142 | 147 | 133 | 156 | 131 | 117 | 141 |
